# Experimental Investigation of Steel Bar Corrosion in Recycled Plastic Aggregate Concrete Exposed to Calcium Chloride Cycles

**DOI:** 10.3390/ma18143361

**Published:** 2025-07-17

**Authors:** Federica Zanotto, Alice Sirico, Andrea Balbo, Patrizia Bernardi, Sebastiano Merchiori, Vincenzo Grassi, Beatrice Belletti, Cecilia Monticelli

**Affiliations:** 1 Department of Engineering, Corrosion and Metallurgy Study Centre “A. Daccò”, University of Ferrara, Via G. Saragat 4A, 44122 Ferrara, Italy; andrea.balbo@unife.it (A.B.); sebastiano.merchiori@unife.it (S.M.); vincenzo.grassi@unife.it (V.G.); 2 Department of Engineering and Architecture, University of Parma, Parco Area delle Scienze 181/A, 43124 Parma, Italy; alice.sirico@unipr.it (A.S.); patrizia.bernardi@unipr.it (P.B.); beatrice.belletti@unipr.it (B.B.)

**Keywords:** recycled plastics, compressive strength, corrosion, chlorides, reinforced concrete

## Abstract

Recycling plastics waste into concrete represents one of the possible approaches for its valorization, offering both economic and environmental benefits. Although numerous studies have explored the mechanical properties of concrete with plastics waste, its durability performance remains largely unexplored. In this context, this study aims to assess the electrochemical behavior of rebars embedded in reinforced concrete modified by partially replacing natural aggregates with recycled plastics, comparing their behavior to that of conventional concrete. The corrosion of reinforcing steel bars was evaluated by wet and dry cycles (w/d) in calcium chloride solutions, monitoring corrosion potential and potentiostatic polarization resistance, and recording electrochemical impedance spectroscopy (EIS) and polarization curves. In addition, the chloride diffusion tendency and the mechanical performances were assessed in unreinforced samples. The findings indicate that in environments with lower chloride concentrations, concrete with plastic granules provides good protection against rebar corrosion. Although the mechanical results of the studied mixes confirmed that incorporating plastic granules as aggregates in the concrete matrix causes a reduction in compressive strength, as known in the literature, the modified concrete also exhibits improved post-cracking behavior, resulting in enhanced ductility and fracture toughness.

## 1. Introduction

Concrete demand is constantly increasing, especially in developing countries; for this reason, reducing the environmental footprint of its production is one of the most challenging tasks that the concrete industry is facing in the 21st century [1,2,3,4,5]. The environmental impact of concrete production can be reduced by minimizing the use of Portland cement through the use of supplementary cementitious materials, especially those represented by industrial by-products, and by finding alternatives to the incorporation of quarry aggregates, substituting them with aggregates derived from waste materials [2,6]. For example, efforts are being made to use coarse aggregates derived from construction and demolition waste, and dredged sands and mining wastes as fine aggregates [3].

In this context, the use of recycled plastics as partial replacement for natural aggregates has been explored by several authors in the literature [7,8,9,10,11,12,13,14,15,16,17], with the aim of finding the second application for post-consumer recycled plastics, which, generally, is lower quality and has less uniform properties than virgin material [1], thus helping solve the issues related to plastic waste disposal [11,18]. Moreover, advantages can be achieved in terms of reduced use of quarry aggregates and energy savings related to their subsequent treatments (washing, grinding, and transport) [3].

Different types of recycled plastic materials were introduced into concrete as a partial replacement of natural aggregates. For example, many research works regarded the use of fine aggregate manufactured from recycled waste polyethylene terephthalate (PET) [10,12,19,20,21,22], which was added in different amounts, shapes [8,17,20], and sizes [10,21], and in some cases, their surface was modified with other materials [19,21], to improve the adhesion between aggregate surfaces and concrete interface. In other research activities, the replacement of conventional aggregates was obtained with recycled high- (HDPE) or low-density polyethylene (LDPE) [15,21,23,24,25] and recycled polypropylene (PP) [15,21,26] or polypropylene fibers [9,17].

In the preceding studies, it was shown that recycled plastic waste in concrete influences several properties of fresh and hardened concrete, such as density, air content, workability, compressive and tensile strength, modulus of elasticity, impact resistance, permeability, and abrasion resistance [11]. As expected, as the content of plastic aggregates increases, a decrease in fresh and hardened density is generally observed [11,27,28] because of the lower density of plastic materials with respect to natural aggregates. On the contrary, workability results vary widely depending on the size, shape, and type of plastic particles [11,12,19,27], particularly when they are added to replace sand [22,27,29]. Few data are reported on the air void content; in particular, a negligible increase was observed when up to 20% of sand was replaced by fine plastics [25]. It is suggested that air bubbles may form at the interface between plastic aggregates and cement due to the hydrophobic nature of plastics [30].

Most research works have shown a gradual decrease in compressive strength as the percentage of recycled waste plastics, replacing both coarse and fine aggregates, increases [27]. Similar trends have been observed with other mechanical properties, such as splitting tensile strength and elastic modulus [11,31,32,33]. This reduction in strength has been attributed to the inferior strength and stiffness of plastic aggregates themselves with respect to natural ones and to the scarce adhesion and increased stress at the interfacial transition zone (ITZ) between the plastics and cementitious matrix, which in turn may be related to the formation of air voids within the matrix [21,22,24,29,34]. Plastics hydrophobicity explains the presence of ITZ discontinuities and air voids in concrete [30]. Since the poor adhesion between the plastic particles and the cement matrix represents the major obstacle to the use of recycled plastics in concrete, some techniques have been proposed to improve bond properties [1]. However, these treatments are expensive and lead to increased energy consumption and costs, making them less viable at an industrial scale. Recently, a study [35] highlights the possibility of including other kinds of waste materials able to counterbalance the reduction in compressive strength.

Despite these drawbacks, some mechanical advantages have been noted. In terms of impact resistance and energy absorption, some studies [36,37,38] have suggested an improvement when the plastic aggregates are added, as the inherent flexibility of plastics can enhance the concrete’s ability to absorb energy, by delaying the crack initiation and slowing down crack propagation. Hence, the performance in terms of impact resistance and energy absorption tends to stably increase with increasing levels of substitution of both fine and coarse aggregates, making concrete incorporating plastic materials particularly attractive for structural applications requiring high energy absorption, such as in cases where dynamic loads or impact resistance are critical for structural safety.

The durability of concrete containing recycled plastic aggregates is particularly important to guarantee the protective effect of the concrete against the steel rebars and thus delay their corrosion initiation as much as possible. Some researchers [10,24,26], through water absorption and porosity tests, studied the capability of these concretes to protect steel rebars from corrosion. They generally found that water absorption increases when the percentage of natural aggregates replaced by plastics is significantly increased. However, in some studies [39,40], the results of the rapid chloride ion permeability test indicate that permeability decreases when plastic aggregates are inserted in the concrete mixtures. This effect was attributed by Kou et al. [41] to the impermeable nature of plastics waste, which presents a physical obstacle to chloride diffusion.

Concerning the electrochemical behavior of rebars embedded in concrete containing recycled plastics waste, very few research studies are present in the literature. For example, Gavela et al. [14], performed electrochemical tests on reinforced concrete samples in which natural aggregates were partially substituted with industrial wastes from polypropylene (PP) or high-density polyethylene (HDPE) (12% by volume replacement of fine conventional aggregates), during partial and complete immersion in 3.5 % NaCl solution. They concluded that the partial replacement of natural aggregates by the two studied polymers did not worsen the corrosion behavior of reinforcing bars.

As shown, several studies can be found in the literature that have primarily focused on the mechanical properties of concrete containing recycled plastics; however, the durability aspects—particularly the corrosion resistance of embedded steel reinforcement—have remained largely unexplored. To further investigate the corrosion resistance of steel rebars, the research activity presented in this work primarily was focused on the study of the electrochemical behavior of reinforced concrete (RC) samples in which the recycled plastic grains were added to the admixture by replacing 13% and 20% (by volume) of natural aggregates. These samples were exposed to chloride solutions in wet/dry conditions, and their durability and mechanical behavior were compared, according to standard specifications [42,43,44,45], to that of a conventional reinforced concrete.

The experimental program, therefore, can represent a pioneering contribution in the context of durability of concrete incorporating plastic waste, offering novel insights into how the recycled plastics influence the corrosion performance of concrete in chloride-rich environments. These findings are not only relevant from a sustainability perspective to achieve the internationally established goals (such as Agenda 2030 SDGs and EU Green Deal objectives), but also provide an important scientific basis for the disciplines of Civil Engineering, Materials Engineering, and Environmental Engineering, supporting the broader adoption of recycled plastics in construction applications with greater confidence in their long-term performance.

## 2. Materials and Methods

### 2.1. Concrete Production and Preparation of Specimens

All the concrete samples were prepared using a recommended recipe representing a traditional concrete for structural use as a reference batch, designated in the following as CEM. The other two mixes, named P13 and P20, were obtained by replacing 13% and 20% (by volume) of natural aggregates with recycled plastic granules, as can be seen in Table 1.

The cement used was Type II A-LL 42.5R, with a specific gravity of 3.07 and a Blaine fineness of 3900 cm^2^/g. The chemical tests, performed according to EN 196-2:2013 [46] and UNI 10595:1997 [47], report the content of SO_3_ equal to 2.8%, Cl equal to 0.09, and C_3_A equal to 8.5.

Locally available calcareous sand and siliceous gravel were used for casting. The corresponding particle size distributions are detailed in Table 2. For the reference concrete (CEM), fine and coarse aggregates were used at a weight ratio of 2.75:1.375:1 relative to the cement powder.

The recycled plastic granules used as partial aggregate replacement were derived from industrial packaging waste and regranulated into roughly lentil-shaped particles (1–2 mm thick and 3–4 mm in diameter). Due to this non-homogeneous composition of low-density polyethylene (LDPE) and polyamide (PA), with PA ranging from 25 to 75 by weight, this material is generally disposed of in landfills.

All the concrete mixes were prepared with a water-to-cement ratio equal to 0.5, while the superplasticizer dosage (Mapei Dynamon Xtend W202R, MAPEI S.p.A., Milan, Italy) was varied to obtain about the same slump value (about 200 mm-class S4) for the three mixes. In detail, 50% and 62% less superplasticizer was required for P13 and P20, respectively, with respect to CEM. This effect, which is linked to the hydrophobic nature of plastic granules [35], suggests that the substitution of part of natural aggregates with recycled plastics can enhance the concrete flowability, leading to lower costs or improved strength and durability when the water content is reduced, instead of reducing the superplasticizer.

Mixing was performed in a standard drum-type mixer, following the same mix sequence for standard concrete. Plastic granules, along with the natural aggregates and half of the water, were added first. Cement, the remaining water, and the superplasticizer were added in the final phase. The resulting concrete was homogeneous, with no signs of segregation or bleeding.

Different molds were used during the casting phase to produce specific samples for mechanical and electrochemical tests.

In more detail, the experimental program outline for both mechanical tests and the study of the corrosion behavior is presented in Table 3 and Table 4, respectively.

**Figure 1:**
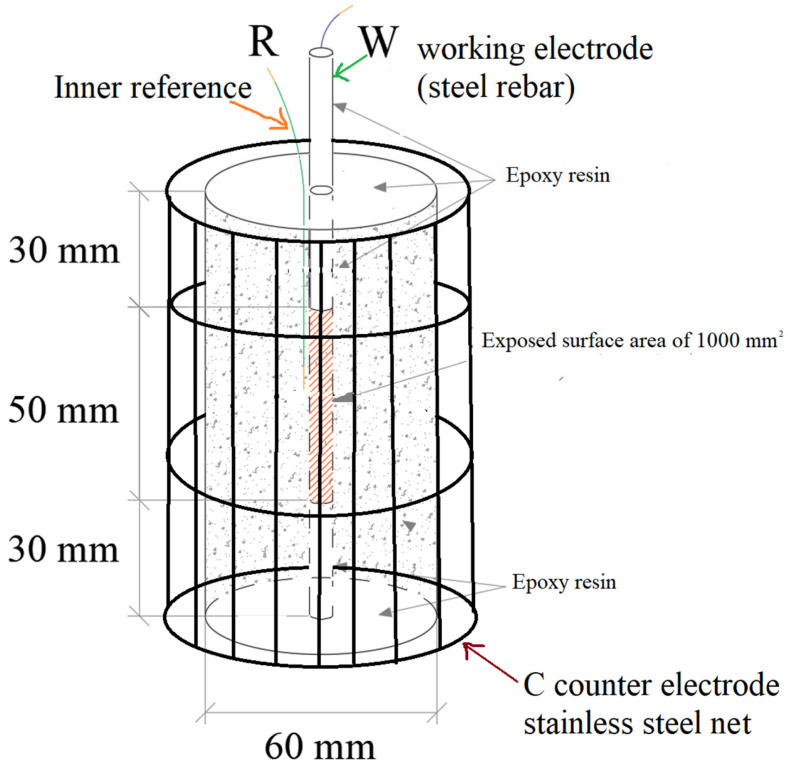
Sample and configuration (working electrode/reference electrode/counter electrode) used for the electrochemical tests.

Figure 1 shows the sample features and configuration (working electrode—W; reference electrode—R; counter electrode—C) used for the electrochemical tests, which were similar to those used in previous research activities [48]. The 6 mm diameter central rebars (acting as working electrode) were in sandblasted steel B450C. The rebar surfaces were covered with an epoxy resin, with the exception of a surface area of about 1000 mm^2^. The Ti inner reference electrode was placed in the corresponding area.

The chloride content evaluation and pH assessments were carried out on unreinforced samples with the same shape and dimensions as the reinforced ones.

All the samples produced were cured in the molds for one day before being removed and then cured under standard conditions (i.e., in water) for the first 28 days and finally tested or subjected to wet/dry exposure.

### 2.2. Mechanical Tests

The density was determined by measuring both the volume of three cubic samples for each mix, as well as their mass, by following EN 12390-7 [49] recommendations.

The compressive strength *f_c_* and splitting tensile strength *f_ct_*_,*sp*_ were determined on three specimens for each mix and exposure condition by following the procedure detailed in EN 12390-3 [44] and EN 12390-6 [45], respectively. A METROCOM PV P30 Universal Testing Machine (Metrocom Engeneering, Piedmont, Italy) was used as the scope by applying a loading rate of 0.5 MPa/s and 0.05 MPa/s for compressive and tensile tests, respectively.

The flexural strength and fracture energy were determined on at least three notched samples for each mix and exposure condition using an Instron 8862 testing machine (Instron, Norwood, MA, USA) and following JCI-S-001-2003 [50] recommendations. Before testing, at mid-span, a notch 2 mm wide and 30 mm deep (i.e., equal to 30% beam depth) was cut by a concrete saw.

The notched beams were then tested over a net span *S* of 300 mm by applying a three-point bending configuration and using a clip gauge to perform the tests under Crack Mouth Opening Displacement (CMOD) control. The initial rate was chosen equal to 0.6 mm/h until the peak load *P_max_* was reached, and then the displacement rate was increased until the end of the test, which corresponded to a residual load of 0.015 kN. The test set-up allowed providing the complete load (P)-CMOD curve until failure for each sample. Starting from this curve, both flexural strength *f_ct_*_,*fl*_ as well as the post-cracking behavior, through fracture energy *G_f_* evaluation were obtained.

The flexural strength *f_ct_*_,*fl*_ was evaluated as

(1) $$f_{ct,fl}=P_{\max}\frac{3S}{2BH^{2}}$$

where *B* (equal to 100 mm) and *H* (equal to 70 mm) are the width and the net depth of the notched mid-cross section.

Fracture energy *G_f_* was evaluated as

(2) $$G_{f}=\frac{0.75\;W_{0}+W_{1}}{A_{lig}}$$

where *W*_0_ is related to the area under the P-CMOD curve, *W*_1_ indicates the work performed by the specimen deadweight and loading equipment, and *A_lig_* is the area of the ligament (equal to *B × H*).

### 2.3. Chloride Exposure

After a period of standard curing (28 days) in water, the samples were exposed to 4 days of immersion in CaCl_2_ and Ca(OH)_2_ solutions and 3 days of drying under laboratory conditions. After 1 year of exposure to these conditions, on unreinforced cubic, cylindrical, and prismatic samples, mechanical tests were carried out. The Cl^−^ ion concentration in the solution was 0.2 M (0.1 M CaCl_2_ solution) in the first 84 days (12 w/d cycles), and then the exposure continued with a 0.6 M Cl^−^ concentration (0.3 M CaCl_2_ solution) up to one year.

Concerning the cylindrical reinforced samples (designed for electrochemical tests) before the exposure to the same conditions as the unreinforced ones, their upper and lower surfaces were found to have chloride diffusion only in the radial direction. During the exposure, after a precise number of cycles, the phenolphthalein test was adopted to assess carbonation depth on sections of the unreinforced samples. At the same time, pH measurements were carried out by mixing 3 g of powder with 3 cm^3^ of distilled water. The powder was obtained by drilling the innermost concrete portion of the unreinforced samples (i.e., the one corresponding to the concrete in contact with the rebar in the reinforced samples). Moreover, standard methods [39,40] were applied to determine free and total chloride concentrations (as wt% vs. binder) in the concrete powder at different distances from the central axis of the cylindrical samples.

### 2.4. Electrochemical Tests

During each cycle, electrochemical measurements, with a 273A PAR instrument (Ametek, Berwyn, PA, USA), were performed to evaluate the corrosion behavior of the rebar embedded in reinforced samples. Corrosion potential (E_cor_) values were measured both versus the Ti inner electrode and versus an external electrode (a saturated calomel, SCE). The linear polarization resistance technique was used to determine the polarization resistance (R_p_) values. A small voltage (+10 mV vs. E_cor_) was applied for a small time (300 s). The ratio of voltage to current is the polarization resistance value, and, according to the Stern–Geary relationship [51,52], is inversely proportional to the corrosion current given by the expression i_cor_ = B/R_p_, where B is a constant of 26 mV for both steel rebar in passive and active or corroded state [53]. The ohmic drop between the rebar and the Ti reference electrode was always negligible with respect to R_p_ values (as confirmed by electrochemical impedance spectroscopy (EIS)). Moreover, EIS spectra were acquired (with Solartron EI 1287, FRA 1260 (Ametek, Berwyn, PA, USA) combined with Zview 3.5g software) by applying, starting from E_cor_, a ±10 mV fluctuation in the frequency range spanning from 10^5^ to 10^−^^3^ Hz.

At selected times of exposure, cathodic and anodic polarization curves were recorded, with ohmic compensation applied, from E_cor_, applying a scanning rate of 0.166 mVs^−^^1^. When the acquired electrochemical parameters indicated a constant active state of the reinforcement bars (i.e., E_cor_ lower than −0.350 mV_SCE_ and i_cor_ higher than 0.2 µA/cm^2^), the extent and the morphology of the corrosion attack were observed by opening the reinforced samples.

## 3. Results and Discussion

### 3.1. Mechanical Characterization

**Figure 2:**
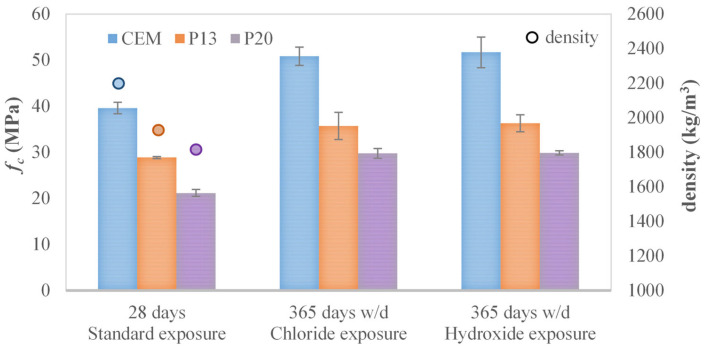
Average compressive strength *f_c_* (and corresponding standard deviation) for CEM, P13, and P20 after 365 days of w/d exposure, together with 28-day compressive strength and density measurements.

The compressive test results obtained on concrete samples are illustrated in Figure 2 and Table 5 in terms of the average values of compressive strength (*f_c_*) with related standard deviations. After 28 days of standard curing, an approximately linear decrease in compressive strength with the increase in the percentage of plastic aggregates in the concrete can be observed. Reductions of 27% and 47% are obtained for P13 and P20 with respect to CEM, respectively. The same trend can also be observed at 7 days (with reductions equal to 22% and 42% for P13 and P20 with respect to CEM), indicating that the addition of plastics affects the absolute strength values but not their rate of increase over time.

These results are explained by the poor physical/chemical compatibility between the hydrophobic plastic granules and the hydrophilic cement matrix. This behavior is mainly attributable to the loss of bond between the plastics surface and the cement matrix caused by the hydrophobic nature of plastics, which limits cement hydration reaction at the plastic/cement interface [54]. The presence of unabsorbed water creates empty interfaces between the two materials, which increases the porosity of concrete, weakening the interfacial transition zone and lowering the bonding strength. Furthermore, the lower elastic modulus and hardness of plastic granules compared to natural aggregates play an important role in reducing compressive strength, as already highlighted in the literature [14]. For structural concrete applications, this reduction in compressive strength can represent one of the main challenges in recycling plastics as aggregates in concrete. However, research in this field is still under development. As an example, previous studies showed that a possible improvement of mechanical strengths is possible by combining, in concrete mix design, plastic aggregates with other kinds of waste materials able to counterbalance the decrease in compressive strength, such as the addition of biochar used as a filler [35].

As can be seen in Figure 2, the same decreasing trend can be recognized for density as well. This is attributable to the lower specific gravity of plastic granules (that is equal to 0.868 g/cm^3^) with respect to natural aggregates (2.64 g/cm^3^). The obtained values (equal to 1933 kg/m^3^ and 1834 kg/m^3^) allow classifying P13 and P20 as lightweight concrete (class D2.0), according to Eurocode 2 [55] classification.

After 365 days of w/d cycles in CaCl_2_ solution, irrespective of the composition of the tested samples, no negative effects in terms of durability can be recognized from compressive strength, as demonstrated by comparing the results after CaCl_2_ and Ca(OH)_2_ exposure reported in Figure 2. Moreover, by comparing the results obtained for the different series, the same trend with respect to 28-day standard curing can be recognized between CEM, P13, and P20 samples exposed to one year of w/d exposure in CaCl_2_ and Ca(OH)_2_ solutions. In more detail, for both chloride and hydroxide exposure, a reduction of 29% and 42% can be seen for P13 and P20, respectively, with respect to CEM.

**Figure 3:**
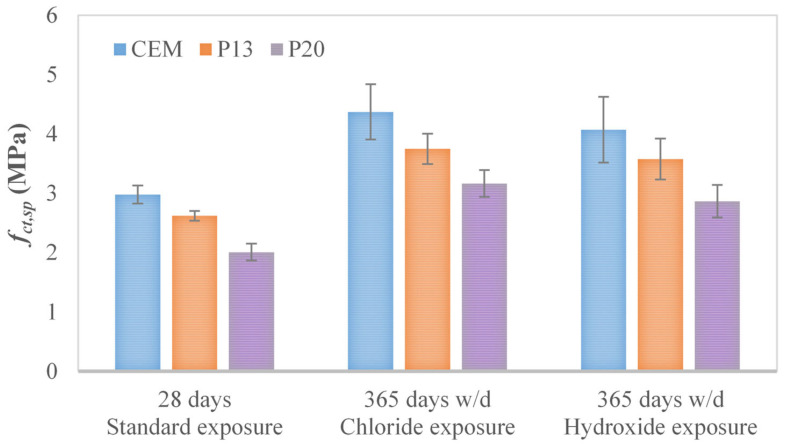
Average splitting tensile strength *f_ct_*_,*sp*_ (and corresponding standard deviation) for CEM, P13, and P20 after 365 days of w/d exposure, together with 28-day measurements.

The splitting test results, shown in Figure 3 and Table 6, indicate that, across all exposure conditions, the use of plastic aggregates has a less detrimental effect on tensile strength compared to compressive strength. The maximum reductions registered for P13 and P20 by considering all the exposure conditions, with respect to CEM, are indeed 14% and 32%, respectively. A potentially less negative impact of plastics on tensile strength with respect to compressive behavior was already reported in the literature [32,56] for concrete containing different types of plastic waste, in the case of standard water curing. The same trend can be observed when considering lightweight concrete, which tends to have higher tensile strength with respect to non-lightweight concrete characterized by the same cubic compressive strength [35].

**Figure 4:**
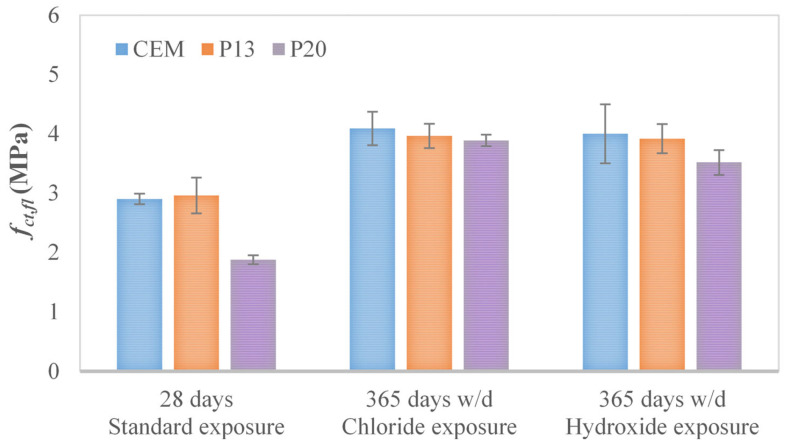
Average flexural tensile strength *f_ct_*_,*fl*_ (and corresponding standard deviation) for CEM, P13, and P20 after 365 days of w/d exposure, together with 28-day measurements.

**Figure 5:**
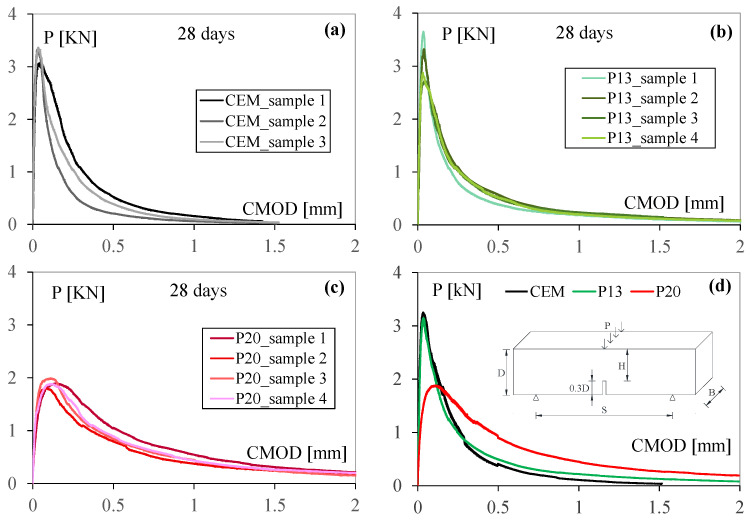
Load P-CMOD curves for (**a**) CEM, (**b**) P13, and (**c**) P20 samples subjected to 28 days of water curing, together with their average values and test sketch (**d**).

The different influence of plastic granules on tensile behavior, with respect to compressive one, is also confirmed by the values of flexural tests reported in Figure 4 and Table 7. For example, as shown in Figure 5, the results of flexural tests after 28 days of water curing, where flexural strength is derived from the peak of the load P-CMOD curve, according to Equation (1). In more detail, after 28 days of water curing, the flexural strength of P13 can be considered equivalent to that of the reference mix (exhibiting a slight increase of approximately 2%), while a reduction of 35% can be seen for P20 with respect to CEM.

In addition, in this case, as already highlighted for the compressive and splitting tensile strength, after one year of w/d cycles in a chloride environment, no damage can be stated for flexural strength due to the aggressive agents. With respect to CEM, a reduction equal to 3% and 5% can be recognized for P13 and P20 for chloride exposure, whereas the reduction is equal to 2% and 12%, respectively, when the samples were exposed to hydroxide solution.

**Figure 6:**
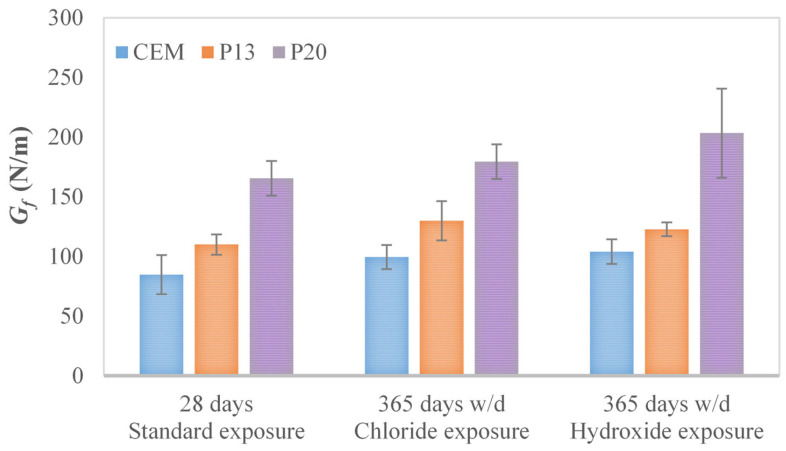
Average fracture energy *G_f_* (and corresponding standard deviation) for CEM, P13, and P20 after 365 days of w/d exposure, together with 28-day measurements.

The fracture energy results, which are illustrated in Figure 5 and Figure 6 and Table 8, show that the use of plastics enhances the post-cracking behavior for all the different exposure conditions considered. As can be seen from the analysis of Figure 4, increasing plastics contents show greater displacement at failure and higher residual loads in post-peak response. In more detail, with respect to CEM, P13 presents improvements in terms of fracture energy ranging from 18% to 30%, while for P20, improvements range from 80% to 96% (see Figure 6). These improvements are statistically significant across all the exposures, as confirmed by the one-way ANOVA studies conducted at a 5% significance level, indicating a 95% confidence that the observed improvements are attributable to the incorporation of plastic waste.

The enhanced behavior in the post-cracking stage can be related to the elasto-plastic and non-brittle behavior of plastic granules. This ability to absorb energy helps to distribute the stress more evenly within the concrete, thereby delaying crack initiation, slowing down the propagation of micro-cracks, and allowing the concrete to exhibit greater ductility, as also reported in [56]. This leads to higher residual loads and larger displacements at failure, since incorporating flexible plastic granules in concrete contributes to the ductility of the concrete, resulting in a significant improvement in the concrete’s energy absorption capacity and increasing its capacity to endure post-cracking tensile loads, also allowing for a more gradual failure (this was observed both in splitting and flexural tests).

### 3.2. Analytical Measurements During the Exposure

**Figure 7:**
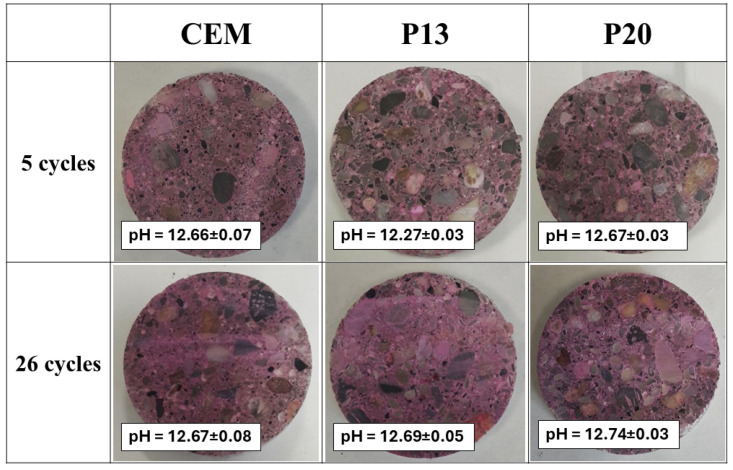
Sections of unreinforced concrete cylinders after 5 and 26 w/d cycles in CaCl_2_ solution: carbonation depth with phenolphthalein and pH measurements.

Figure 7 shows the sections of the unreinforced concrete cylinders, sprayed with phenolphthalein to assess the carbonation depth, after 5 and 26 w/d cycles in CaCl_2_ solution. The images indicate the absence of carbonation during the exposure, as also evidenced by the pH measurements of powders from the central portion of the cylinder section. The pH values remained for all concrete samples around 12.5 throughout the exposure period, indicating passive conditions around the rebars.

**Figure 8:**
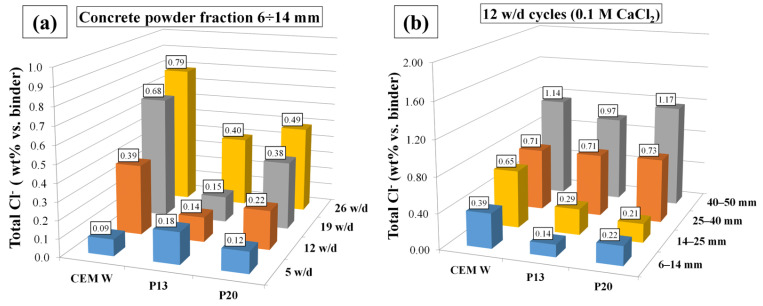
Mean total chloride (wt% vs. binder) (**a**) at 5, 12, 19, and 26 w/d cycles in concrete powder fraction between 6 and 14 mm diameters, and (**b**) after 12 w/d cycles in different concrete powder fractions (diameters ranging between 6 and 50 mm).

**Figure 9:**
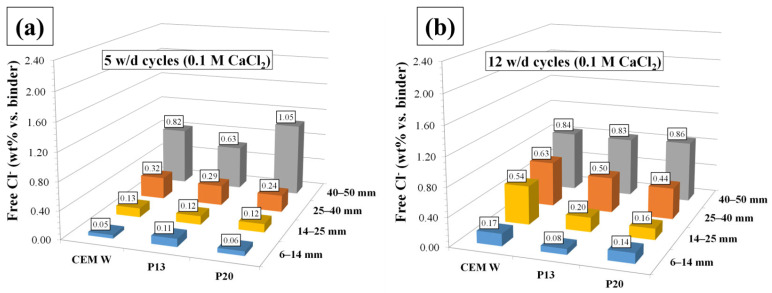
Mean free chloride (wt% vs. binder) at 5 (**a**) and 12 (**b**) w/d cycles in the concrete powder fractions ranging between 6 and 50 mm from the sample axis.

The mean total chloride contents (wt% vs. binder) at different exposure times, measured in the concrete powder fraction between 6 and 14 mm, are reported in Figure 6. This fraction corresponds to the closest concrete powder to the rebars in the electrochemical samples. At the beginning of the exposure, the amount of chlorides that reached the concrete/rebar interface was higher for the plastic-containing samples compared to the CEM samples. However, for longer exposure times, chloride diffusion tendency in P13 and P20 was lower, likely due to the hydrophobic nature of polymeric aggregates, which contributes to slowing down the capillary water absorption [10] and thus Cl^−^ migration towards the rebar/concrete interface. This is also demonstrated by the amount of total Cl^−^ after 12 w/d cycles detected in the different powder fractions. In the more external fractions (between 25 to 40 mm and 40 to 50 mm), the total Cl^−^ wt% vs. binder was rather similar for P13, P20, and CEM. On the contrary, in the more inner powder fractions, a lower amount of total Cl^−^ was determined. The slight differences between P13 and P20 are likely due to the different plastics granule distributions. Concerning free Cl^−^ wt% vs. binder (Figure 8) after 5 w/d cycles, differences between CEM, P13, and P20 were observable only in the more external powder fractions, while, after 12 w/d cycles (Figure 9), a lower amount of free Cl^−^ wt% was measured in the inner powder fractions (between 6 to 14 mm and 14 to 25 mm) in P13 and P20, compared to CEM.

In spite of the higher porosity in P13 and P20 samples [35], by increasing the w/d exposure time, there is a decreasing tendency for chlorides to migrate towards the bar/concrete interface in comparison to the traditional concrete samples (CEM), likely due to the hydrophobic effect of the plastics and the possible presence of entrapped air at interfacial transition zone, which make the path more impervious [24,25,33,39].

### 3.3. Electrochemical Tests

#### 3.3.1. Linear Polarization Resistance Measurements

**Figure 10:**
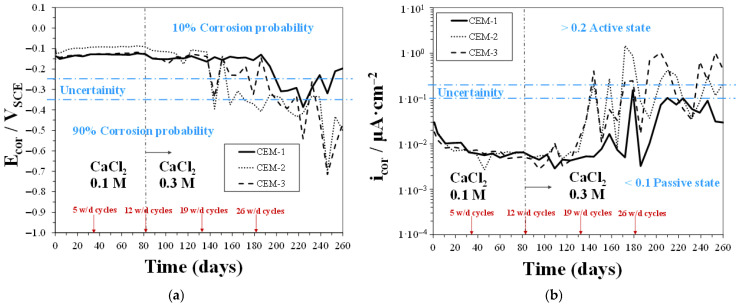
E_cor_ (**a**) and i_cor_ (**b**) vs. time for CEM samples during w/d cycles in solutions with 0.2 M Cl^−^ concentration, up to 12 cycles, and then with 0.6 M Cl^−^ concentration.

**Figure 11:**
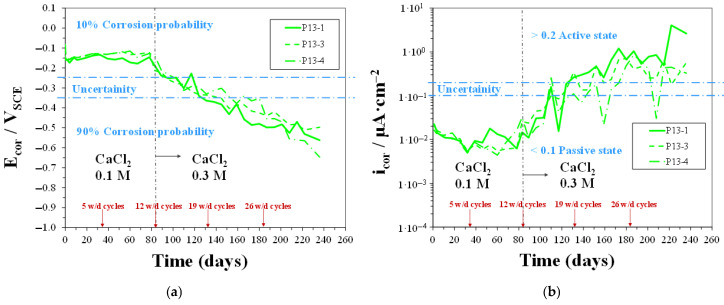
E_cor_ (**a**) and i_cor_ (**b**) vs. time for P13 samples during w/d cycles in solutions with 0.2 M Cl^−^ concentration, up to 12 cycles, and then with 0.6 M Cl^−^ concentration.

**Figure 12:**
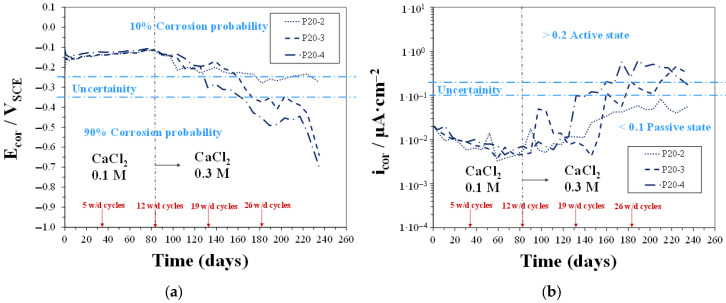
E_cor_ (**a**) and i_cor_ (**b**) vs. time for P20 samples during w/d cycles in solutions with 0.2 M Cl^−^ concentration, up to 12 cycles, and then with 0.6 M Cl^−^ concentration.

In Figure 10, Figure 11 and Figure 12, time trends of E_cor_ and i_cor_ values were measured for CEM, P13, and P20 samples, respectively, during w/d cycles in solutions with 0.2 M Cl^−^ concentration (0.1 M CaCl_2_), up to 12 cycles, and then with 0.6 M Cl^−^ concentration (0.3 M CaCl_2_). In the mildly concentrated Cl^−^ solution (during the first 12 w/d cycles), all the CEM samples showed a clear passive behavior, as already discussed in a preceding work [48]. In the same environmental conditions, concrete containing plastic waste, i.e., both P13 and P20 samples, showed E_cor_ and i_cor_ values, evidence that rebars maintained a passive behavior. E_cor_ ranged between −0.2 and −0.1 V_SCE_ for both P13 and P20, and i_cor_ for P20, starting from about 0.02 µA/cm^2^, decreased, after the 12 w/d cycles, to values around 0.006 µA/cm^2^, in agreement with CEM samples’ behavior. In P13, i_cor_ values were more fluctuating and reached, after 12 cycles, higher values, close to 0.03 µA/cm^2^. These values were about one order of magnitude lower than the threshold, indicating active corrosion in concrete steel reinforcements [53].

When the Cl^−^ ion concentration was increased to 0.6 M (i.e., after the 12th w/d cycle), with the aim of accelerating chloride diffusion, the CEM samples showed a clear tendency to maintain the passive behavior up to the 19th cycle. A different trend was observed for concrete containing plastic waste. In particular, P13 samples showed E_cor_ values quickly shifting towards the more negative values: around −0.35 V_SCE_ at 19 w/d cycles and between −0.5 and −0.65 V_SCE_ at 34 w/d cycles, indicating a 90% probability for active corrosion. In fact, at the same time, i_cor_ quickly increased to values higher than 0.2 µA/cm^2^. For P20 samples, the shifting of E_cor_ towards values indicating active corrosion was slower: At 19 w/d cycles, E_cor_ was still around −0.2 V_SCE,_ and i_cor_ was under 0.1 µA/cm^2^. However, at longer exposure times, two out of three samples exhibited E_cor_ values gradually shifting towards −0.6/−0.7 V_SCE_ and i_cor_ increasing to values around 0.3 µA/cm^2^. This behavior indicated a slower and more gradual weakening of passive conditions in the rebars embedded in P20 than those in P13 samples. Moreover, it can be observed, up to 19 cycles, that the behavior of P20 was quite close to that of the CEM samples.

#### 3.3.2. Electrochemical Impedance Spectroscopy

**Figure 13:**
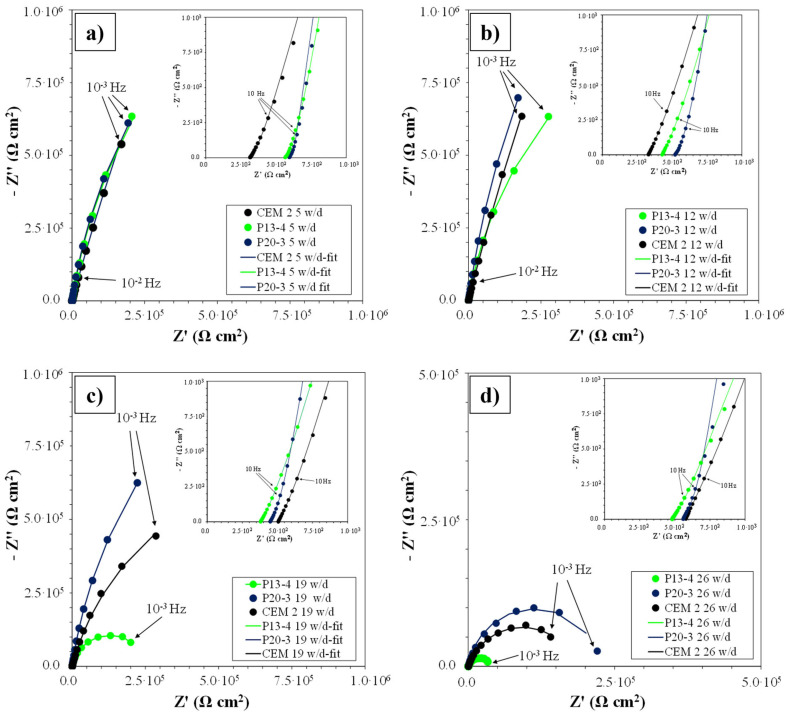
EIS spectra (experimental data as symbols and simulated data as continuous line) recorded on CEM 2, P13-4, and P20-3 after 5 w/d (**a**), 12 w/d (**b**), 19 w/d (**c**), and 26 w/d cycles (**d**) in 0.2 M Cl^−^ solution, up to 12 cycles, and then in 0.6 M Cl^−^ solution.

Figure 13 shows EIS spectra (reported as Nyquist plot), recorded on CEM and plastic-added samples, during w/d cycles in solutions with 0.2 M Cl^−^ concentration (0.1 M CaCl_2_), up to 12 cycles (a and b), and then in solutions with 0.6 M Cl^−^ concentration (0.3 M CaCl_2_) (c and d). After 5 w/d cycles (Figure 13a), very similar Nyquist plots were observed. After 12 cycles (Figure 13b), P13-4 spectra, at low frequencies, showed the tendency to close and form a semicircle, thus indicating a decrease in polarization resistance value, Rp, in comparison to that of P20-3 and CEM-2 samples. This tendency was more evident after 19 cycles (Figure 13c) for all the samples. In particular, the R_p_ value (determined as the intercept of the low frequency part of the semicircle with the real axis of the Nyquist plot) of about 2.5·105 Ω·cm^2^ was measured for P13-4, corresponding to a value of corrosion current density of 0.1 μA/cm^2^. After 26 cycles, the measured R_p_ from EIS spectra were about 3.9 × 10^4^, 2.3 × 10^5^, and 1.8 × 10^5^ Ω·cm^2^ for P13-4, P20-3, and CEM-2, respectively.

All the spectra presented two capacitive arcs corresponding to two time constants in the Bode plot, indicating that the partial substitution of traditional aggregates with recycled plastics in concrete does not result in any modification of the EIS spectra when compared to those of traditional concrete. The first capacitive loop, at frequencies higher than 10^1^ Hz (shown in the boxes of Figure 13a–d), was very small and ill-resolved, because it was overlapping with the low frequencies’ capacitive arc, detectable between 10^2^ and 10^−3^ Hz. The capacitive loop at low frequencies is frequently attributed to the corrosion product layer on the rebar [57,58] or to the presence of a cementitious film on the rebar surface, with its own characteristics different from mortar bulk [59,60]. Since the time constant at high frequencies is already present during the first cycles, when the rebars are still in passive conditions, this constant is possibly connected to the dielectric properties of steel/concrete interface [61], while the second capacitive arc between 10^2^ and10^−3^ Hz is ascribed to charge transfer reactions on the rebar surface.

An equivalent circuit (EC), already studied in the literature [58,62,63], was used for fitting the acquired EIS spectra. The EC is composed of two elements with a resistance (R) and a constant phase element (CPE) in parallel and preceded by a resistance R_s+m_, taking into account electrolyte filling the pores and concrete resistance between the inner reference Ti electrode and the steel surface. The experimental data, reported in the diagrams as symbols, are presented together with the fitting results curves, reported as a continuous line in Figure 13. The R_f_-CPE_f_ parallel element fits the high frequencies arc, associated with the dielectric properties of the steel/concrete interface, while the R_ct_-CPE_dl_ one fits the low frequencies one, giving information about the corrosion process parameters (R_ct_, charge transfer resistance, and C_dl_, double layer capacitance).

The constant phase element, CPE, instead of an ideal capacitance, C, was used in the EC to compensate for surface inhomogeneities and discontinuities at interfaces [48,64]. In Table 9 and Table 10, the results of EIS spectra fitting with the EC are reported for the P13 and P20 samples. These parameters are compared to those of CEM presented in a previous research [48]. R_s+m_ is influenced by solution conductivity, which may increase due to Cl^‚−^ diffusion through the concrete matrix and by concrete hydration. The reference electrode was placed in proximity to the exposed area of the rebar, so R_s+m_ values were always quite small, in the range 300 ÷ 700 Ω·cm^2^; however, they generally tended to increase, likely due to the ongoing concrete curing. The R_f_ and C_f_ parameters, linked to the h_f_ time constant, presented slightly increasing values likely linked to the progress of concrete hydration, determining an increase in the rebar surface covered by the film of mortar at the steel/concrete interface.

R_ct_ values were lower than those of CEM-2 [48] since the beginning of the exposure, in particular for P13, evidencing a lower tendency to form a stable passive film on the rebar surface if compared to CEM. In more detail, for P20-3, R_ct_ increased up to 12 w/d cycles, indicating the formation of a passive layer, but starting from 19 cycles, in the more Cl^−^ concentrated solution, a decrease was detected. In the P13-4 sample, the tendency for the R_ct_ to decrease was observed already after 12 w/d cycles.

#### 3.3.3. Polarization Curves

**Figure 14:**
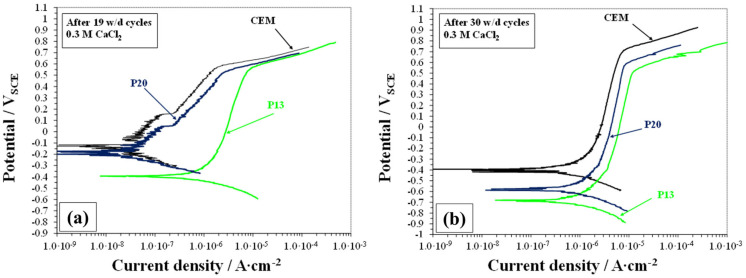
Polarization curves recorded on CEM, P13, and P20 samples after (**a**) 19 w/d cycles and (**b**) 30 w/d cycles in 0.3 M CaCl_2_ solution.

The polarization curves recorded on reinforcing bars in CEM, P13, and P20 samples, after 19 and 30 w/d cycles, are presented in Figure 14.

At the beginning of the exposure to the more chloride concentrated solution, rebars in CEM samples were under stable passive conditions, as described in [48,65]. The same behavior was displayed by the rebar in P20, showing an E_cor_ value around −0.180 V_SCE_ and very low i_cor_, close to 0.01 µA/cm^2^ (thus under 0.1 µA/cm^2,^ indicating a passive behavior). In contrast, in the P13 sample, rebars showed a less passive behavior, characterized by E_cor_ values around −0.380 V_SCE_ and pseudopassive currents higher than 0.2 µA/cm^2^. At potentials higher than +0.6 V_SCE_, all samples showed a transpassive behavior with current density increasing rapidly with potential [66]. As already observed in [48], after 30 w/d cycles, the pseudopassive behavior of rebars in CEM samples (E_cor_ and i_cor_ values) indicated the presence of moderate corrosion conditions [67]. After the same exposure time, rebar in the P13 and P20 samples showed a very negative E_cor_ value (close to −0.700 and −0.600 V_SCE_, respectively) and quite higher corrosion rates (i_cor_ around 0.8 and 0.4 µA/cm^2^, respectively) compared to CEM, in particular for P13.

Overall, in the less concentrated chloride environment (up to 12 w/d cycles), the electrochemical results showed a good tendency of the plastic-added concrete mixes to protect steel rebars from corrosion, and thus to maintain a passive behavior for the reference concrete samples. Subsequently, when the chloride concentration was increased, the electrochemical behavior of steel rebars displayed the tendency to develop corrosion, which is more marked in P13 samples than in P20, likely depending on both the amount and the distribution of recycled plastic granules. The onset of chloride corrosion in reinforced concrete essentially depends on three factors: the presence of an electrolyte; the diffusion of oxygen towards the rebar surface; and the depassivation of the steel due to a critical chloride concentration. The electrolyte is the aqueous solution that fills the concrete’s pores during the wet step of the w/d exposure, allowing chloride diffusion. Oxygen diffusion occurs mainly in the dry step, again through the concrete’s porosity. The chloride content in the concrete near the reinforcement was similar for the two plastics mixes, so the different corrosion initiation times cannot be explained by a different critical chloride content. The difference between the P13 and P20 samples lay in the higher content of plastic granules in the P20 mix, which replaced the coarser aggregates. This increase resulted in higher porosity at the cement/plastics interface, raising the probability that these aggregates would be in close proximity to the reinforcement bar. On the one hand, the increased porosity would lead to a greater amount of solution permeating through the concrete. However, as the plastic granules are hydrophobic, this could reduce the retention of a wet environment around the rebar in sample P20 during the dry step and finally allow the sample to dry more quickly while maintaining passive conditions for longer exposure times.

**Figure 15:**
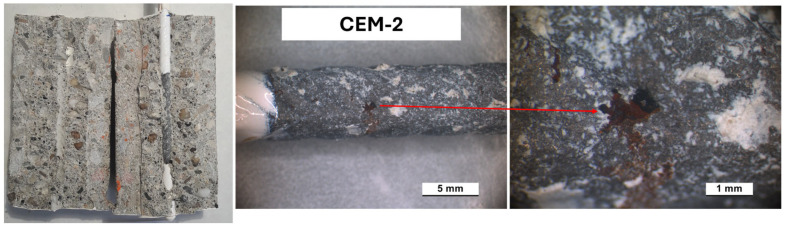
Images after opening the CEM-2 sample at the end of the exposure.

**Figure 16:**
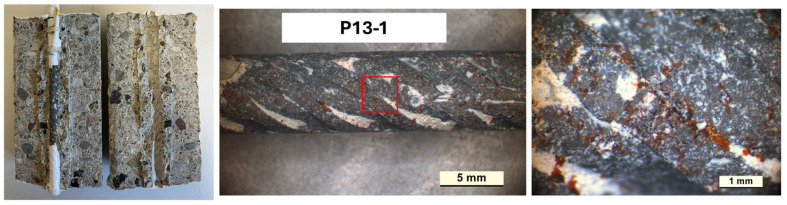
Images after opening the P13-1 sample at the end of the exposure.

**Figure 17:**
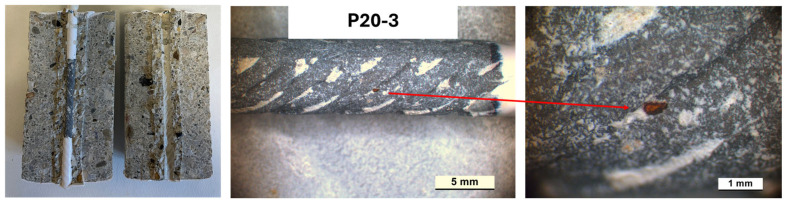
Images after opening the P20-3 sample at the end of the exposure.

An evaluation of the corrosion attack level was carried out, with E_cor_ and i_cor_ values indicating active corrosion conditions on the rebars. All samples were cut and opened. Figure 15, Figure 16 and Figure 17 present photos of the cut sample evidencing a good bond at the rebars/concrete interface and zones in which a localized corrosive attack developed. In the P13-1 sample (Figure 16), the localized attack diffused in a wider portion of the rebar surface during the exposure. This behavior agrees with E_cor_ and i_cor_ trends, evidencing values typical of active conditions for a larger time of exposure compared to CEM-2 and P20-3.

## 4. Conclusions

In this research activity, the effect of partially replacing natural aggregates with recycled plastic granules, coming from regranulation of industrial waste, on the mechanical resistance of concrete and the durability of reinforced concrete samples was assessed. In consideration of the results obtained, the following conclusions can be deduced.

- Partial replacement of natural aggregate with plastic granules results in a decrease in both compressive strength and tensile (splitting and flexural) strength, even if the latter appears less influenced than compressive strength by the addition of plastic granules. In more detail, P13 and P20 show a compressive strength reduction of about 25% and 45%, respectively, compared to CEM, while the tensile strength decreases by approximately 15% and 30%. On the contrary, a significant increase in fracture energy is observed (about 30% for P13 and 95% for P20 compared to CEM), indicating an enhanced capacity to absorb and redistribute tensile forces. These results underline the role of plastic aggregates in transforming the failure mode of concrete, providing valuable insights into improving its brittle behavior.
- Wet and dry exposure to chlorides for 365 days does not adversely affect the mechanical strength of plastic-added concrete.
- The hydrophobic nature of plastics granules resulted in higher concrete flowability. In more detail, the mixtures P13 and P20 required 50% and 62% less superplasticizer, respectively, than CEM to reach the same slump values. Moreover, this same characteristic of plastics allows to slow the tendency to migration of chlorides towards the reinforcement bar, with average total chloride concentrations (after 26 w/d cycles) of 0.40 and 0.49 (wt% vs. binder) for P13 and P20, respectively, compared to what was observed in the reference concrete with a concentration of 0.79 (wt% vs. binder). The amount and distribution of the plastic granules, influencing the porosity of concrete and its ability to retain water in the drying step of the w/d cycle, have an influence on the passivity of the rebar and, consequently, the corrosion development.
- In lower chloride environments, concrete with recycled plastics provides good protection against reinforcing bar corrosion, with i_cor_ values, after 12 w/d cycles, close to 0.006 and 0.03 µA/cm^2^ for P20 and P13, respectively. However, with higher chloride contents, the performance of plastic-added concrete worsens, while remaining within acceptable limits.

The results obtained are overall promising and open a path towards the recycling of plastic waste in concrete to be used in the building industry, promoting environmentally sustainable construction practices, and addressing the challenges posed by plastic waste management. Future research, in particular, could focus on developing mix designs that incorporate recycled plastics together with other waste materials to counteract the negative impact of plastics on compressive strength.

## Figures and Tables

**Table 1:** Mix proportions in kg/m^3^.

Mix	Cement	Sand	Gravel	Plastic Waste	Water	Superplasticizer
CEM	408	1126	562	-	204	3.88
P13	408	900	562	74	204	1.92
P20	408	900	449	111	204	1.47

**Table 2:** Particle size distribution of natural fine and coarse aggregates.

Sieve Size (mm)	Cumulative % Weight Retained	
	Sand	Gravel
10.000	0	0
8.000	0	16.0
5.600	1.1	80.7
4.000	18.2	97.2
2.000	36.6	98.8
1.000	59.3	99.0
0.500	85.5	99.6
0.250	96.7	99.8
0.125	99.1	100
0.063	100.0	100

**Table 3:** Experimental program outline for mechanical behavior analysis.

Test Type	Samples	Specimen Dimensions (mm)
Hardened density	3	150 × 150 × 150
Compressive strength at 7 days of standard curing	3	150 × 150 × 150
Compressive strength at 28 days of standard curing	3	150 × 150 × 150
Compressive strength at 365 days w/d chloride exposure	3	150 × 150 × 150
Compressive strength at 365 days w/d hydroxide exposure	3	150 × 150 × 150
Splitting strength at 28 days of standard curing	3	Φ100 × 200
Splitting strength at 365 days w/d chloride exposure	3	Φ100 × 200
Splitting strength at 365 days w/d hydroxide exposure	3	Φ100 × 200
Flexural strength at 28 days of standard curing	≥3 *	100 × 100 × 400
Flexural strength at 365 days w/d chloride exposure	≥3 *	100 × 100 × 400
Flexural strength at 365 days w/d hydroxide exposure	≥3 *	100 × 100 × 400
Fracture energy at 28 days of standard curing	≥3 *	100 × 100 × 400
Fracture energy at 365 days w/d chloride exposure	≥3 *	100 × 100 × 400
Fracture energy at 365 days w/d hydroxide exposure	≥3 *	100 × 100 × 400

* The number of specimens varies between 3 and 6, depending on the mix series and exposure type.

**Table 4:** Experimental program outline for the corrosion behavior study.

Test Type	Samples	Specimen Dimensions (mm)
Phenolphthalein test for assessing carbonation depth	4	unreinforced cylinders Φ60 × 110
pH measurements of concrete powder	4	unreinforced cylinders Φ60 × 110
Free and total chloride concentrations	4	unreinforced cylinders Φ60 × 110
Corrosion potential measurement and linear polarization resistance (LPR) technique	4	reinforced cylinders Φ60 × 110
Electrochemical impedance spectroscopy (EIS) and polarization curves	4	reinforced cylinders Φ60 × 110

**Table 5:** Compressive test results with the corresponding standard deviations: at 7 days (*f_c_*_,*7*_) and 28 days of standard exposure (*f_c_*_,*28*_), and after 365 days of wet and dry cycle under CaCl_2_ (*f_c_*_,*365*,*Cl*_) and Ca(OH)_2_ exposure (*f_c_*_,*365*,*hydro*_).

Mix	*f_c_*_,*7*_ (MPa)	*f_c_*_,*28*_ (MPa)	*f_c_*_,*365*,*Cl*_ (MPa)	*f_c_*_,*365*,*hydro*_ (MPa)
CEM	33.04 ± 1.34	39.58 ± 1.24	50.79 ± 1.99	51.67 ± 3.33
P13	25.62 ± 0.41	28.81 ± 0.24	35.68 ± 2.95	36.26 ± 1.82
P20	19.23 ± 0.39	21.13 ± 0.75	29.72 ± 1.05	29.82 ± 0.45

**Table 6:** Splitting tensile test results with the corresponding standard deviations: at 28 days of standard exposure (*f_ct_*_,*sp*,*28*_), and after 365 days of wet and dry cycle under CaCl_2_ (*f_ct_*_,*sp*,*365*,*Cl*_) and Ca(OH)_2_ exposure (*f_ct_*_,*sp*,*365*,*hydro*_).

Mix	*f_ct_*_,*sp*,*28*_ (MPa)	*f_ct_*_,*sp*,*365*,*Cl*_ (MPa)	*f_ct_*_,*sp*,*365*,*hydro*_ (MPa)
CEM	2.98 ± 0.15	4.37 ± 0.47	4.07 ± 0.55
P13	2.62 ± 0.08	3.75 ± 0.25	3.57 ± 0.34
P20	2.01 ± 0.14	3.16 ± 0.23	2.86 ± 0.28

**Table 7:** Flexural tensile test results with the corresponding standard deviations: at 28 days of standard exposure (*f_ct_*_,*fl*,*28*_), and after 365 days of wet and dry cycle under CaCl_2_ (*f_ct_*_,*fl*,*365*,*Cl*_) and Ca(OH)_2_ exposure (*f_ct_*_,*fl*,*365*,*hydro*_).

Mix	*f_ct_*_,*fl*,*28*_ (MPa)	*f_ct_*_,*fl*,*365*,*Cl*_ (MPa)	*f_ct_*_,*fl*,*365*,*hydro*_ (MPa)
CEM	2.90 ± 0.09	4.09 ± 0.28	4.00 ± 0.50
P13	2.96 ± 0.30	3.97 ± 0.21	3.92 ± 0.25
P20	1.88 ± 0.08	3.89 ± 0.10	3.52 ± 0.21

**Table 8:** Fracture energy values with the corresponding standard deviations: at 28 days of standard exposure (G*_f_*_,*28*_), and after 365 days of wet and dry cycle under CaCl_2_ (G*_f_*_,*365*,*Cl*_) and Ca(OH)_2_ exposure (G*_f_*_,*365*,*hydro*_).

Mix	G*_f_*_,*28*_ (N/m)	G*_f_*_,*365*,*Cl*_ (N/m)	G*_f_*_,*365*,*hydro*_ (N/m)
CEM	84.7 ± 16.3	99.4 ± 10.2	103.9 ± 10.3
P13	109.9 ± 8.6	129.9 ± 16.5	122.8 ± 5.8
P20	165.5 ± 14.6	179.4 ± 14.5	203.2 ± 37.4

**Table 9:** Parameters obtained with fitting of EIS spectra for P13-4.

Time: w/d Cycles	4	8	12	19	26
E_cor_/V_SCE_	−0.129	−0.158	−0.263	−0.315	−0.373
R_s+m_/Ω cm^2^	346	507	391	379	465
R_f_/Ω cm^2^	68	60	60	110	95
C_f_/µF cm^−2^	154	74	39	98	166
R_ct_/kΩ cm^2^	1360	6129	888	253	38
C_dl_/µF cm^−2^	273	253	244	234	270

**Table 10:** Parameters obtained with fitting of EIS spectra for P20-3.

Time: w/d Cycles	4	8	12	19	26
E_cor_/V_SCE_	−0.187	−0.174	−0.158	−0.178	−0.273
R_s+m_/Ω cm^2^	525	475	520	488	687
R_f_/Ω cm^2^	66	70	98	100	122
C_f_/µF cm^−2^	185	129	53	102	40
R_ct_/kΩ cm^2^	3086	5817	7962	2946	235
C_dl_/µF cm^−2^	274	271	270	239	222

## Data Availability

The original contributions presented in this study are included in the article. Further inquiries can be directed to the corresponding author.

## References

[1] Meyer C. (2009). The Greening of the Concrete Industry. Cem. Concr. Compos..

[2] Juenger M.C.G., Winnefeld F., Provis J.L., Ideker J.H. (2011). Advances in Alternative Cementitious Binders. Cem. Concr. Res..

[3] Kumar P. (2001). Mehta Reducing the Environmental Impact of Concrete. Concrete Can Be Durable and Environmentally Friendly. Concr. Int..

[4] Schneider M. (2019). The Cement Industry on the Way to a Low-Carbon Future. Cem. Concr. Res..

[5] Miller S.A., Horvath A., Monteiro P.J.M. (2016). Readily Implementable Techniques Can Cut Annual CO_2_ Emissions from the Production of Concrete by over 20%. Environ. Res. Lett..

[6] Russo N., Filippi A., Carsana M., Lollini F., Redaelli E. (2025). Impact of RAP as Recycled Aggregate on Durability-Related Parameters of Structural Concrete. Mater. Struct. Constr..

[7] Zanotto F., Sirico A., Merchiori S., Vecchi F., Balbo A., Bernardi P., Belletti B., Malcevschi A., Grassi V., Monticelli C. (2022). Durability of Reinforced Concrete Containing Biochar and Recycled Polymers. Key Eng. Mater..

[8] Belletti B., Bernardi P., Sirico A., Balbo A., Zanotto F. (2022). Experimental Bond Behaviour of Reinforced Concrete With Recycled Plastic Aggregates.

[9] Marzouk O.Y., Dheilly R.M., Queneudec M. (2007). Valorization of Post-Consumer Waste Plastic in Cementitious Concrete Composites. Waste Manag..

[10] Siddique R., Khatib J., Kaur I. (2008). Use of Recycled Plastic in Concrete: A Review. Waste Manag..

[11] Choi Y.W., Moon D.J., Kim Y.J., Lachemi M. (2009). Characteristics of Mortar and Concrete Containing Fine Aggregate Manufactured from Recycled Waste Polyethylene Terephthalate Bottles. Constr. Build. Mater..

[12] Bravo M., De Brito J. (2012). Concrete Made with Used Tyre Aggregate: Durability-Related Performance. J. Clean. Prod..

[13] Saikia N., De Brito J. (2012). Use of Plastic Waste as Aggregate in Cement Mortar and Concrete Preparation: A Review. Constr. Build. Mater..

[14] Gavela S., Ntziouni A., Rakanta E., Kouloumbi N., Kasselouri-Rigopoulou V. (2013). Corrosion Behaviour of Steel Rebars in Reinforced Concrete Containing Thermoplastic Wastes as Aggregates. Constr. Build. Mater..

[15] Hossain M., Bhowmik P., Shaad K. (2016). Use of Waste Plastic Aggregation in Concrete as a Constituent Material. Progress. Agric..

[16] Hou M., Li Z., Li V.C. (2024). Green and Durable Engineered Cementitious Composites (GD-ECC) with Recycled PE Fiber, Desert Sand, and Carbonation Curing: Mixture Design, Durability Performance, and Life-Cycle Analysis. Constr. Build. Mater..

[17] Anto J., Bhuvaneshwari M. (2024). A Comprehensive Summary on the Enhancement of Properties of Concrete with Recycled Polypropylene Materials. Struct. Concr..

[18] Oddo M.C., Cavaleri L., Ferrara L., Muciaccia G., Trochoutsou N. (2025). Plastic Waste for Concrete Mixture: Advanced Strategies and Solutions. Proceedings of the RILEM Spring Convention and Conference.

[19] Choi Y.W., Moon D.J., Chung J.S., Cho S.K. (2005). Effects of Waste PET Bottles Aggregate on the Properties of Concrete. Cem. Concr. Res..

[20] Almeshal I., Tayeh B.A., Alyousef R., Alabduljabbar H., Mohamed A.M. (2020). Eco-Friendly Concrete Containing Recycled Plastic as Partial Replacement for Sand. J. Mater. Res. Technol..

[21] Thorneycroft J., Orr J., Savoikar P., Ball R.J. (2018). Performance of Structural Concrete with Recycled Plastic Waste as a Partial Replacement for Sand. Constr. Build. Mater..

[22] Rahmani E., Dehestani M., Beygi M.H.A., Allahyari H., Nikbin I.M. (2013). On the Mechanical Properties of Concrete Containing Waste PET Particles. Constr. Build. Mater..

[23] Hasan A., Islam M.N., Karim M.R., Habib M.Z., Wahid M.F. Properties of Concrete Containing Recycled Plastic as Coarse Aggregate. Proceedings of the IICSD-2015 International Conference on Recent Innovation in Civil Engineering for Sustainable Development.

[24] Steyn Z.C., Babafemi A.J., Fataar H., Combrinck R. (2021). Concrete Containing Waste Recycled Glass, Plastic and Rubber as Sand Replacement. Constr. Build. Mater..

[25] Chen C.-C., Nathan J., Matt K., Wesley W., Albert P. (2015). Concrete Mixture with Plastic As Fine Aggregate. Int. J. Adv. Mech. Civ. Eng..

[26] Coppola B., Courard L., Michel F., Incarnato L., Scarfato P., Di Maio L. (2018). Hygro-Thermal and Durability Properties of a Lightweight Mortar Made with Foamed Plastic Waste Aggregates. Constr. Build. Mater..

[27] Babafemi A.J., Šavija B., Paul S.C., Anggraini V. (2018). Engineering Properties of Concrete with Waste Recycled Plastic: A Review. Sustainability.

[28] Hameed A.M., Ahmed B.A.F. (2019). Employment the Plastic Waste to Produce the Light Weight Concrete. Energy Procedia.

[29] Ismail Z.Z., AL-Hashmi E.A. (2008). Use of Waste Plastic in Concrete Mixture as Aggregate Replacement. Waste Manag..

[30] Jacob-Vaillancourt C., Sorelli L. (2018). Characterization of Concrete Composites with Recycled Plastic Aggregates from Postconsumer Material Streams. Constr. Build. Mater..

[31] Guo Y.C., Li X.M., Zhang J., Lin J.X. (2023). A Review on the Influence of Recycled Plastic Aggregate on the Engineering Properties of Concrete. J. Build. Eng..

[32] Bahij S., Omary S., Feugeas F., Faqiri A. (2020). Fresh and Hardened Properties of Concrete Containing Different Forms of Plastic Waste—A Review. Waste Manag..

[33] Almeshal I., Tayeh B.A., Alyousef R., Alabduljabbar H., Mustafa Mohamed A., Alaskar A. (2020). Use of Recycled Plastic as Fine Aggregate in Cementitious Composites: A Review. Constr. Build. Mater..

[34] Islam M.J., Meherier M.S., Islam A.K.M.R. (2016). Effects of Waste PET as Coarse Aggregate on the Fresh and Harden Properties of Concrete. Constr. Build. Mater..

[35] Sirico A., Bernardi P., Sciancalepore C., Belletti B., Milanese D., Malcevschi A. (2023). Combined Effects of Biochar and Recycled Plastic Aggregates on Mechanical Behavior of Concrete. Struct. Concr..

[36] Tayeh B.A., Almeshal I., Magbool H.M., Alabduljabbar H., Alyousef R. (2021). Performance of Sustainable Concrete Containing Different Types of Recycled Plastic. J. Clean. Prod..

[37] Saxena R., Siddique S., Gupta T., Sharma R.K., Chaudhary S. (2018). Impact Resistance and Energy Absorption Capacity of Concrete Containing Plastic Waste. Constr. Build. Mater..

[38] Al-Tayeb M.M., Aisheh Y.I.A., Qaidi S.M.A., Tayeh B.A. (2022). Experimental and Simulation Study on the Impact Resistance of Concrete to Replace High Amounts of Fine Aggregate with Plastic Waste. Case Stud. Constr. Mater..

[39] Cotto-Ramos A., Dávila S., Torres-García W., Cáceres-Fernández A. (2020). Experimental Design of Concrete Mixtures Using Recycled Plastic, Fly Ash, and Silica Nanoparticles. Constr. Build. Mater..

[40] Alani A.H., Bunnori N.M., Noaman A.T., Majid T.A. (2019). Durability Performance of a Novel Ultra-High-Performance PET Green Concrete (UHPPGC). Constr. Build. Mater..

[41] Kou S.C., Lee G., Poon C.S., Lai W.L. (2009). Properties of Lightweight Aggregate Concrete Prepared with PVC Granules Derived from Scraped PVC Pipes. Waste Manag..

[42] (2011). Standard Test Method for Chemical Analysis of Hydraulic Cement.

[43] (2020). Standard Test Method for Water Soluble Chloride in Mortar and Concrete.

[44] (2019). Testing Hardened Concrete—Part 3: Compressive Strength of Test Specimens.

[45] (2009). Testing Hardened Concrete—Part 6: Tensile Splitting Strength of Test Specimens.

[46] (2013). Method of Testing Cement—Part 2: Chemical Analysis of Cement.

[47] (1997). Sulphate-Resistant and Leaching-Resistant Cements. Determination of the Strength Class. Chemical Testing Method.

[48] Zanotto F., Sirico A., Balbo A., Bernardi P., Merchiori S., Grassi V., Belletti B., Malcevschi A., Monticelli C. (2024). Study of the Corrosion Behaviour of Reinforcing Bars in Biochar-Added Concrete under Wet and Dry Exposure to Calcium Chloride Solutions. Constr. Build. Mater..

[49] (2019). Testing Hardened Concrete—Part 7: Density of Hardened Concrete.

[50] (2003). Method of Test for Fracture Energy of Concrete by Use of Notched Beam.

[51] Andrade C., Alonso C. (1996). Corrosion Rate Monitoring in the Laboratory and On-Site. Constr. Build. Mater..

[52] Kanemitsu T., Takaya S. (2023). Applicability of AC Impedance Method for Measuring Time-Variant Corrosion Rate to Cracked and Crack-Repaired Reinforced Concrete. Mater. Struct..

[53] Andrade C. (2019). Propagation of Reinforcement Corrosion: Principles, Testing and Modelling. Mater. Struct. Constr..

[54] Bhagat G.V., Savoikar P.P. (2022). Durability Related Properties of Cement Composites Containing Thermoplastic Aggregates—A Review. J. Build. Eng..

[55] (2015). Eurocode 2–Design of Concrete Structures—Part 1–1: General Rules and Rules for Buildings.

[56] Khaleel Y.U., Qubad S.D., Mohammed A.S., Faraj R.H. (2024). Reinventing Concrete: A Comprehensive Review of Mechanical Strength with Recycled Plastic Waste Integration. J. Build. Pathol. Rehabil..

[57] Aperador W., Duque J., Delgado E. (2016). Comparison of Electrochemical Properties between Portland Cement, Ground Slag and Fly Ash. Int. J. Electrochem. Sci..

[58] Yu F., Chen M., Zhou M., Yang Q., Xie H., Yin H., Li W., Poon C.S., Liu F. (2025). Study on Corrosion Characteristics of Steel Rebars and Corrosion-Induced Cracks in Reinforced Concrete by Employing X-Ray Microcomputed Tomography. J. Build. Eng..

[59] Montemor M.F., Simoes A.M.P., Salta M.M., Ferreira M.G.S. (1993). The Assessment of the Electrochemical Behaviour of Flyash-Containing Concrete by Impedance Spectroscopy. Corros. Sci..

[60] Keddam M., Takenouti H., Nóvoa X.R., Andrade C., Alonso C. (1997). Impedance Measurements on Cement Paste. Cem. Concr. Res..

[61] Gu P., Elliott S., Beaudoin J.J., Arsenault B. (1996). Corrosion Resistance of Stainless Steel in Chloride Contaminated Concrete. Cem. Concr. Res..

[62] Feliu V., González J.A., Andrade C., Feliu S. (1998). Equivalent Circuit for Modelling the Steel-Concrete Interface. I. Experimental Evidence and Theoretical Predictions. Corros. Sci..

[63] Criado M., Martínez-Ramirez S., Fajardo S., Gõmez P.P., Bastidas J.M. (2013). Corrosion Rate and Corrosion Product Characterisation Using Raman Spectroscopy for Steel Embedded in Chloride Polluted Fly Ash Mortar. Mater. Corros..

[64] Monticelli C., Zanotto F., Balbo A., Grassi V., Fabrizi A., Timelli G. (2022). Corrosion Behavior of High-Pressure Die-Cast Secondary AlSi9Cu3(Fe) Alloy. Corros. Sci..

[65] Monticelli C., Natali M.E., Balbo A., Chiavari C., Zanotto F., Manzi S., Bignozzi M.C. (2016). A Study on the Corrosion of Reinforcing Bars in Alkali-Activated Fly Ash Mortars under Wet and Dry Exposures to Chloride Solutions. Cem. Concr. Res..

[66] Volpi E., Olietti A., Stefanoni M., Trasatti S.P. (2015). Electrochemical Characterization of Mild Steel in Alkaline Solutions Simulating Concrete Environment. J. Electroanal. Chem..

[67] Aguirre-Guerrero A.M., Robayo-Salazar R.A., Mejía de Gutiérrez R. (2021). Corrosion Resistance of Alkali-Activated Binary Reinforced Concrete Based on Natural Volcanic Pozzolan Exposed to Chlorides. J. Build. Eng..

